# Effectiveness of motivational interviewing, health education and brief advice in a population of smokers who are not ready to quit

**DOI:** 10.1186/s12874-018-0511-0

**Published:** 2018-06-13

**Authors:** Majid Bani-Yaghoub, Abdellatif Elhomani, Delwyn Catley

**Affiliations:** 1 Department of Mathematics and Statistics, University of Missouri–Kansas City, 5100 Rockhill Road, Kansas City, MO 64110-2499 USA; 2 School of Medicine, Creighton University, 2500 California Plaza, Omaha, NE 68178 USA; 3 Department of Pediatrics, Center for Children’s Healthy Lifestyles & Nutrition, 2401 Gillham Road, Kansas City, MO 64108 USA; 4 Children’s Mercy Hospitals and Clinics, University of Missouri-Kansas City School of Medicine, 2401 Gillham Road, Kansas City, MO 64108 USA

**Keywords:** Smoking cessation, Markov chain model, Motivational interviewing, Brief advice, Health education, Efficacy, Effectiveness

## Abstract

**Background:**

Motivational Interviewing (MI), Brief Advice (BA) and Health Education (HE) are established smoking cessation induction methods for smokers with low desire to quit. Although randomized controlled trials (RCT’s) have been frequently used to assess these interventions the *temporal efficacy* and *effectiveness* of these interventions have been poorly elaborated. The present work endeavors to fill the gap by considering the full range of possible motivational outcomes for *all* of the participants.

**Methods:**

As a two-step process, Markov Chain (MC) and Ordinary Differential Equation (ODE) models were successively employed to examine the temporal efficacy and effectiveness of these interventions by computing the gradual movements of participants from an initial stage of unmotivated smoker to stages of increased motivation to quit and cessation. Specifically, in our re-analysis of data from the RCT we examined the proportion of participants in 4 stages of readiness to quit (unmotivated, undecided, motivated, former smokers) over 6 months, across treatment groups [MI (*n* = 87), BA (*n* = 43) and HE (*n* = 91)].

**Results:**

Although HE had greater efficacy compared to MI and BA (i.e., the highest smoking cessation rates), it had lower effectiveness at certain time points. This was due to the fact that HE had the greatest proportion of motivated smokers who quit smoking but simultaneously a large proportion of the motivated smokers became unmotivated to quit. The effectiveness of HE dropped substantially in weeks 3–12 and remained below the effectiveness of BA from week 12 onward. The 2-year ODE model projections show that the prevalence of motivated smokers in HE group may fall below 5%. The prevalence of HE former smokers can reach an equilibrium of 26%, where the prevalence of both BA and MI former smokers exceeds this equilibrium.

**Conclusions:**

The methodology proposed in this paper strongly benefits from the capabilities of both MC and ODE modeling approaches, in the event of low observations over the time. Particularly, the temporal population sizes are first estimated by the MC model. Then they are used to parametrize the ODE model and predict future values. The methodology enabes us to determine and compare the temporal efficacy and effectiveness of smoking cessation interventions, yielding predictive and analytic insights related to temporal characteristics and capabilities of these interventions during the study period and beyond.

**Trial registration:**

Testing Counseling Styles to Motivate Smokers to Quit, NCT01188018, (July 4, 2012). This study is registered at www.clinicaltrials.gov NCT01188018.

**Electronic supplementary material:**

The online version of this article (10.1186/s12874-018-0511-0 contains supplementary material, which is available to authorized users.

## Background

Traditional methods of evaluating smoking cessation treatments often involve conducting randomized trials and assessing one of the outcomes (i.e., quit attempts, motivation, and cessation) at a particular point in time, such as the middle or the end of the follow-up period [1–3].

Statistical analyses such as the Pearson chi-square test and logistic regression are useful for studying the effects of smoking cessation interventions at a particular time point [4, 5]. These approaches appear to be suitable when the smoking cessation is viewed as a unidirectional problem. However smoking cessation is a process that takes place over time, not a single event occurring at a certain time [6]. Therefore, analysis of smoking cessation data requires alternative approaches that are beyond the scope of single time-point methodologies. Smoking cessation is increasingly recognized as a process of change subject to fluctuations in motivation, attempts to quit, periods of abstinence, and often relapse [7, 8]. Applying single time-point methods discounts this time-varying nature of smoking cessation. To tackle this issue, researchers have proposed the use of generalized estimating equations models (GEE) and generalized linear mixed-effects models (GLMM) [9]. GLMM estimates the probability that an individual would be either an abstinent or a smoker at a given time point, allowing different partialities of abstinence among individuals. GEE provides estimates for the population average and the covariate effects, which can be interpreted as in normal logistic regression models. As mentioned in [10], GEE and GLMM are efforts to go beyond “time naïve” evaluations of outcomes. However, these two techniques are intrinsically unidirectional. In other words, they measure abstinence (or smoking) across time from smoking at baseline. Alternatively, Markov Chain (MC) models (also known as transition models) offer a means to concurrently examine transitions in both directions from abstinent to smoking and vice versa [10]. These models offer the ability to recognize factors that may work in different directions over time. Identifying factors that are associated with cessation and those with relapse will enable researchers to propose more effective interventions for both smoking cessation and relapse prevention.

MC models have been widely employed to study various time-varying clinical situations including smoking cessation [10–12]. It has been previously demonstrated [10] that MC models can use information in randomized smoking cessation trials that the GEE and GLMM do not exploit, and consequently they can provide additional outcomes. These outcomes include information about the dynamics associated with relapse to smoking among those who are temporarily abstinent as well as the reasons connected with abstinence. In addition, MC models deliver knowledge about transitions between abstinence and relapse in both directions. Other studies have combined Markov modeling with the transition models such as GEE or GLMM [10, 12].

To assess the efficacy and the effectiveness of smoking cessation induction methods, we propose a new methodology that consists of two steps. First, a multi-stage MC model [11, 12] is employed to describe the process of smoking cessation, in which individuals move through a series of stages (i.e., unmotivated, indecisive, motivated and former smoker) in continuous time. Then the outcomes of the fitted MC model are used to parametrize a system of Ordinary Differential Equations (ODE) and to predict and compare the behaviors of individuals participating in different interventions.

Several interventions are currently available [7, 13]. However, Motivational Interviewing (MI) is the only intervention recommended for unmotivated smokers by the U.S. Clinical Practice Guidelines [7]. For clarity, MI is defined as a collaborative, person-centered form of counseling to elicit and strengthen motivation for change [14]. The existing evidence suggests the effect of MI on smoking cessation is likely to be modest [15]. Particularly, meta-analyses have indicated that MI-based interventions have modest positive effects on smoking cessation compared to the other interventions such as Brief Advice (BA) [16–18]. In addition, significant deficiencies have been noted in the evidence base [7, 16, 17]. This includes the inadequate evidence of intervention fidelity, the insufficient research comparing MI to alternative interventions of equal intensity, the lack of focus on initial motivation to quit and absence of studies focusing on motivation and quit attempts as outcomes [16, 17].

The present study focuses on the secondary analysis of a randomized trial to address the limitation of MI research. We carried out the secondary analysis by comparing the efficacy and effectiveness of MI with those of BA and the other intervention known as Health Education (HE) [19]. BA was considered to imitate the usual care based on the recommendations of the Clinical Practice Guideline [7]. In the original study [20], the participants in BA group met with a counselor for approximately 5 min and they were asked about the common smoking related symptoms and provided with clear and personalized advice to quit. The participants in HE group were provided health education and matched in duration with MI intervention. This method of motivating HE participants was according to a robust rationale for quitting covering the relevant risks of smoking, benefits of quitting, and addressing the barricades to quitting [7].

The purpose of the present study was to go beyond the single time-point methods to examine the performance of MI relative to BA and HE for inducing quit attempts among low motivated smokers. Specifically, using the available data and a two-step MC-ODE modeling technique, we measured the temporal changes in the 7-day point-prevalence smoking abstinence and motivational behaviors and compared the efficacy and effectiveness of MI relative to BA and HE. Furthermore, the asymptotic dynamics of quit motivations and smoking abstinence were investigated through the ODE model predictions.

## Methods

### Overview

To study the treatment effects of HE, MI and BA, we considered both probabilistic and deterministic modeling approaches. First, using the self-assessment data [20], the participants of each group were divided into subgroups of unmotivated, indecisive, motivated and former smokers, represented by stages 1–4, respectively. Then a multi-stage MC model [11, 12] was fitted to the data of all 4 stages. Temporal variations in the prevalence of each stage were used to determine the temporal effectiveness and efficacy [21–23] of each intervention. Then an ODE model was specified and used to predict the prevalence of each subgroup for 2 years.

### Data

Participants with missing data were not considered in this study (i.e., 15.6, 14.71 and 10.8% of participants in BA, MI and HE groups respectively were excluded). Excluding those with missing data, there was a total of 43, 87 and 91 participants in BA, MI and HE groups, respectively. Using the following scheme, each group was divided into four subgroups: unmotivated (Stage 1); indecisive (Stage 2); motivated (Stage 3); and former smokers (Stage 4). To determine if an individual was in Stages 1–3, we used the responses to the item *“How motivated are you to quit smoking?”* for weeks 0, 12 and 26. Each individual was considered in Stage 1 if the answer was 0–3; Stage 2 if the answer was 4–7; and Stage 3 if the answer was 8–10. To determine if an individual was in Stage 4, we used the response to the item “*Have you smoked at least part of a cigarette in the past 7 days?”* Regardless of the answer to the first question, an individual was considered in Stage 4 if the answer to the last question was “No”. Hence, Stage 4 represents the 7-day point-prevalence smoking abstinence. See Additional file 1: Table S1 for a summary of the observed number of transitions between the stages. Note that there were two cases in each of MI and HE groups, which were excluded from the data due to relapse. Particularly, relapses occurring from Stage 4 to other stages were not considered in the MC modeling.

### The MC model

**Fig. 1:**
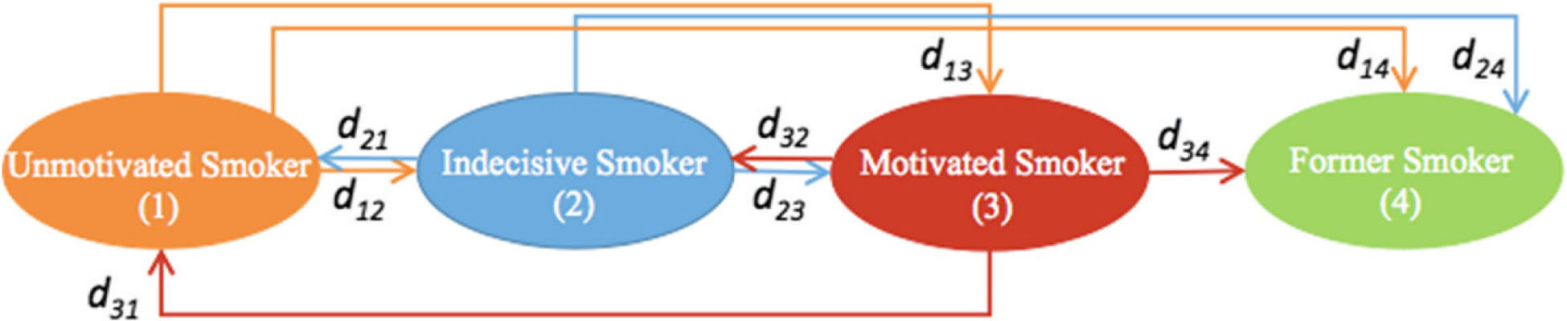
The compartmental diagram of quit progression among smokers who are not ready to quit. The self-assessment data was used to determine the stages of smokers. The arrows show the possible transitions between the stages. Note that the last stage is the 7-day point-prevalence smoking abstinence, which is an absorbing state. The parameters d_ij, denote the rate of transition from stage i to stage j with i,j = 1,…,4;i ≠ j

In our randomized trial, the participants were all smokers at enrollment (*t* = 0) and had two follow-ups with time intervals of 13 weeks. Let *S*_*it*_ be the smoking stage of the *i*^*th*^ participant at time t, which takes values 1–4 if the stage of smoker is “unmotivated”, “indecisive”, “motivated” and “former”, respectively. This has been shown in Fig. 1, which is a compartmental diagram of quit progression among the smokers with transition rate d_ij_, from the stage i to stage j. The *first-order continuous time MC model* has two main assumptions [24]:

1. The Markov property: The future status of smoker depends only on the present and not on the past history of observations, i.e. Pr(*S*_*it*_| *S*_*it* − 1_, *S*_*it* − 2_, ⋯, *S*_*i*0_) = Pr(*S*_*it*_ | *S*_*it* − 1_ ).
2. The stationary transition process: The transition probabilities do not change over time, i.e. Pr(*S*_*it*_ = *k*| *S*_*it* − 1_ = *l*) = Pr(*S*_*iu*_ = *k*| *S*_*iu* − 1_ = *l* ) = *q*_*lk*_ for any *u* ≠ *t*.

Then the MC model associated with Fig. 1 is described by the *transition intensity matrix Q* = [*q*_*lk*_] which is given by

1 $$ Q=\left[\begin{array}{cccc}-\left({q}_{12}+{q}_{13}+{q}_{14}\right)& {q}_{12}& {q}_{13}& {q}_{14}\\ {}{q}_{21}& -\left({q}_{21}+{q}_{23}+{q}_{24}\right)& {q}_{23}& {q}_{24}\\ {}{q}_{31}& {q}_{32}& -\left({q}_{31}+{q}_{32}+{q}_{34}\right)& {q}_{34}\\ {}0& 0& 0& 0\end{array}\right]. $$

Note that each row of *Q* sums to 0; therefore, the diagonal entry *q*_*rr*_ has been replaced with the sum ${q}_{ll}=-\sum \limits_{k=1,l\ne k}^4{q}_{lk}$. The last row is related to Stage 4, former smoker, which is an “absorbing” state. As mentioned earlier, the two relapse cases were excluded from the data.

The MC model was fitted to the data to compute the efficacy and effectiveness of the interventions and to compare the movement patterns of participants between the Stages 1–4. Specifically, we used the “*MSM R package”* [11, 12] to compute the maximum likelihood estimates and the transition intensity matrix. The *transition probability matrix P*(*t*) = [*p*_*lk*_] is a time varying matrix, whose entry *p*_*lk*_ is the probability of being in Stage k at a future time t + u, given the Stage at time u is l, i.e. *p*_*lk*_ = Pr(*S*_*it* + *u*_ = *k*|*S*_*iu*_ = 1). Then *P*(*t*) is calculated using the Kolmogorov relationship *P*(*t*) = *Exp*(*tQ*), where *Q* is the transition intensity matrix [24]. Using the fitted MC model, the prevalence of each subgroup was calculated for the time interval [0, 26].

### Efficacy versus effectiveness

Efficacy and effectiveness are both important measures for evaluating smoking cessation interventions (see for example [21–23]). Nevertheless, the distinction between these two measures is often poorly understood [21, 25]. The efficacy can be defined as the performance of an intervention regardless of any potential side effects, whereas the effectiveness takes into account the negative side effects such as loss of motivation or confidence to quit smoking. We used the following formula to evaluate the relative efficacy of HE, BA and MI

2 $$ \sigma (t)=\frac{F(t)}{F_{max}},\kern20.25em $$

where *F*(*t*) is the prevalence of former smokers at time t and *F*_*max*_ is the maximum prevalence of former smokers in all three intervention groups. To evaluate the relative effectiveness, we applied a penalty according to the increases and decreases in the prevalence of unmotivated and motivated smokers, respectively. In particular, the effectiveness at time *t* was calculated by

3 $$ \rho (t)=\frac{\left(\Delta  F(t)-\alpha \left(\Delta  U(t)-\Delta  M(t)\right)\right)}{N_{max}},\kern8em $$

where *α* ∈ (0, 1) is the penalty; *N*_*max*_ is the maximum value of the numerator among the three interventions; and *∆U*(*t*) and *∆M*(*t*) are the change in prevalence of unmotivated and motivated smokers at time t, respectively.

### The ODE model

To further analyze the dynamics of quit progression we employed an ODE modeling approach. In particular, we formulated the movement of participants using the following ODE model

4 $$ {\displaystyle \begin{array}{l}{U}^{\hbox{'}}(t)={d}_{21}I(t)+{d}_{31}M(t)-\left({d}_{12}+{d}_{13}+{d}_{14}\right)U(t)\\ {}{I}^{\hbox{'}}(t)=-\left({d}_{23}+{d}_{24}+{d}_{21}\right)I(t)+{d}_{32}M(t)+{d}_{12}U(t)\\ {}{M}^{\hbox{'}}(t)={d}_{23}I(t)-\left({d}_{34}+{d}_{32}+{d}_{31}\right)M(t)+{d}_{13}U(t)\end{array}} $$

where U(t), I(t) and M(t) are the proportions of unmotivated, indecisive and motivated smokers, respectively. As shown in Fig. 1, the parameter d_ij_, denotes the rate of transition from stage i to stage j, with *i*, *j* = 1, …, 4; *i* ≠ *j*. Since the total population size is constant, the proportion of former smokers F(t) is obtained by solving system (4) and using

5 $$ \mathrm{F}\left(\mathrm{t}\right)=100-\left(U(t)+I(t)+M(t)\right).\kern8.5em $$

When the system (4) is not under-determined, it only has the trivial equilibrium

6 $$ \left({U}^{\ast },{I}^{\ast },{M}^{\ast },{F}^{\ast}\right)=\left(0,0,0,100\right),\kern11em $$

which is globally stable. When the system is under-determined there are infinitely many equilibria and the stability of the trivial equilibrium (6) is lost (see the Additional file 1, for more details).

The average prevalence of each subgroup was calculated using the formula

7 $$ \kern6em {P}_{avg}=\frac{1}{T}{\int}_0^TP(t) dt,\kern10.25em $$

To estimate the parameters d_ij_ of system (4), the Matlab program “*fmincon.m”* from the optimization toolbox was used. The main idea is to minimize the least squares error between a solution of the ODE model (4) and the set of data points [26, 27]. Here, the prevalence data generated from the MC model was employed to estimate the parameter values of the ODE model (4) for each group of BA, HE and MI participants. Note that the program “*fminsearch.m”* may give rise to negative parameter values, which are not realistic. This was prevented by using “*fmincon.m”* and setting the lower bounds of the parameters equal to zero. After estimating the parameter values, the specified ODE model was used to predict the dynamics of quit progression over 2 years and to determine the asymptotic behavior of the ODE model.

## Results

### Descriptive statistics

For BA, MI and HE, the dataset consisted of 86, 177 and 184 transitions between stages 1–4, respectively (see Additional file 1 (part A)). The temporal changes in the prevalence of each stage are summarized in Table 1. The changes at week 26 with respect to week 0 shows that HE had the highest increase in the prevalence of former smokers. Nevertheless, instead of assessing a single outcome at the end of the follow-up period, the proposed MC-ODE modeling estimates the weekly changes in the prevalence of all stages during the entire follow-up period. This can reveal the dynamics and possible downsides of each intervention during weeks 0 to 26.

**Table 1:** Summary of the observed data in stages of unmotivated, indecisive, motivated and former smokers

Week	Unmotivated	Indecisive	Motivated	Former
Brief Advice (*n* = 43)				
0	31(72.1%)	12(27.9%)	0(0%)	0(0%)
12	20(46.5%)	16(37.2%)	7(16.3%)	0(0%)
26	18(41.9%)	12(27.9%)	10(23.3%)	3(7.0%)
0 → 26	−13(−30.2%)	0(0%)	10(23.3%)	3(7.0%)
Motivational Interviewing (*n* = 87)				
0	69(79.3%)	14(16.1%)	4(4.6%)	0(0%)
12	36(40.9%)	23(26.1%)	27(30.7%)	2(2.3%)
26	32(36.4%)	27(30.7%)	22(25.0%)	7(7.9%)
0 → 26	−37(− 42.9%)	13(14.6%)	18(20.4%)	7(7.9%)
Health Education (*n* = 91)				
0	67(73.6%)	24(26.4%)	0(0%)	0(0%)
12	30(33.0%)	22(24.2%)	33(36.3%)	6(6.6%)
26	23(25.3%)	31(34.1%)	21(23.1%)	16(17.6%)
0 → 26	−44(−48.3%)	7(7.7%)	21(23.1%)	16(17.6%)

Note: The symbol 26 → 0 denotes the changes between weeks 0 and 26

### Analysis of the MC model

Table 2 summarizes the average sojourn time (i.e., average duration of one-time occupancy of a stage), standard error (SE) and 95% confidence intervals related to each intervention. In all three groups, the confidence intervals are wide, which is a limitation of the present study. This could be due to small group sizes [MI (*n* = 87), HE (*n* = 91) and BA (*n* = 43)]. Assuming shorter sojourn time in MI group, the movements of MI participants between the stages (i.e., unmotivated, indecisive, and motivated) could be more frequent than the movements in BA and HE groups. Nonetheless, this is only a speculation and additional data with larger group sizes is needed to confirm this result.

**Table 2:** Average sojourn time (excursion time), standard error (SE), and 95% lower and upper bounds associated with the MC model. For motivational interviewing, the sojourn time was much shorter than the other two interventions

	Estimates	SE	Lower	Upper
Motivational Interviewing (MI)				
unmotivated	2.1041	2.7413	0.1637	27.0420
indecisive	0.4441	1.0043	0.0053	37.3495
motivated	0.4704	1.1268	0.0044	51.4695
Health Education (HE)				
unmotivated	15.883	2.3698	11.8558	21.2784
indecisive	9.6538	4.2429	4.0793	22.8459
motivated	7.7423	2.7391	3.8702	15.4884
Brief Advice (BA)				
unmotivated	10.4213	2.6934	6.2796	17.2948
indecisive	8.3947	2.6351	4.5375	15.5309
motivated	24.1047	14.0375	7.6984	75.4752

Note: The confidence intervals were calculated by simulating 1000 random vectors from the asymptotic multivariate normal distribution implied by the maximum likelihood estimates of the log transition intensities

**Fig. 2:**
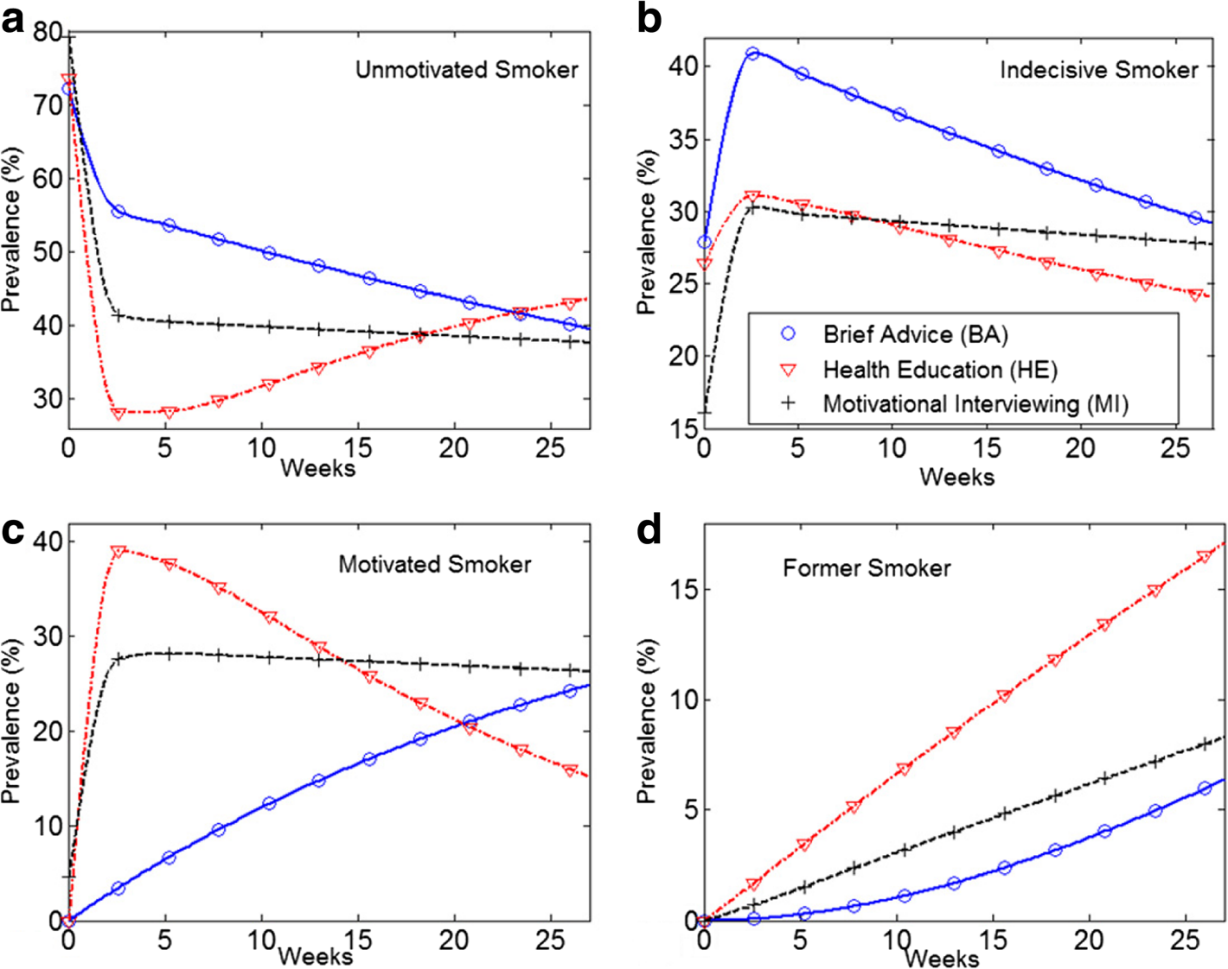
Estimated 26-weeks prevalence using the MC model. **b** BA participants had a higher prevalence of indecisive smokers. **d** Although HE was more effective than MI and BA, HE participants substantially lost their motivations (**c**), many of whom became fully unmotivated (**a**)

The estimated transition intensity matrices *Q* are provided in Table A2 of the Additional file 1. These matrices were used to calculate the estimated 26-weeks prevalence of unmotivated, indecisive, motivated and former smokers in each group. In particular, as shown in panel (b) of Fig. 2, BA participants had a higher prevalence of indecisive smokers. Panel (c) shows that the prevalence of motivated smokers sharply declined in HE group. The decline was accompanied with an incline in the prevalence of unmotivated smokers in HE group (see panel (a)). Nevertheless, as shown in panel (d), HE participants had constantly higher prevalence of former smokers.

**Fig. 3:**
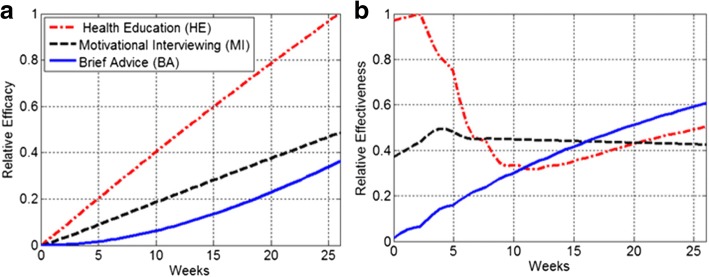
Relative efficacy and effectiveness of BA, HE and MI, **a** The efficacy of HE was superior to BA and MI throughout the study. **b** The effectiveness of HE dropped drastically in weeks 3–12 and it remained below the effectiveness of BA from week 12 onward. A penalty of α = 0.20 was considered in the effectiveness formula (3)

Using the formulas (2) and (3) the relative efficacy and effectiveness of all three interventions were calculated. We applied a penalty of α = 0.20 in the effectiveness formula. As shown in panel (a) of Fig. 3 the efficacy of HE was superior to BA and MI throughout the weeks 0–26. Nevertheless, panel (b) shows that the effectiveness of HE dropped drastically in weeks 3–12 and it remained below the effectiveness of BA from week 12 onward.

**Fig. 4:**
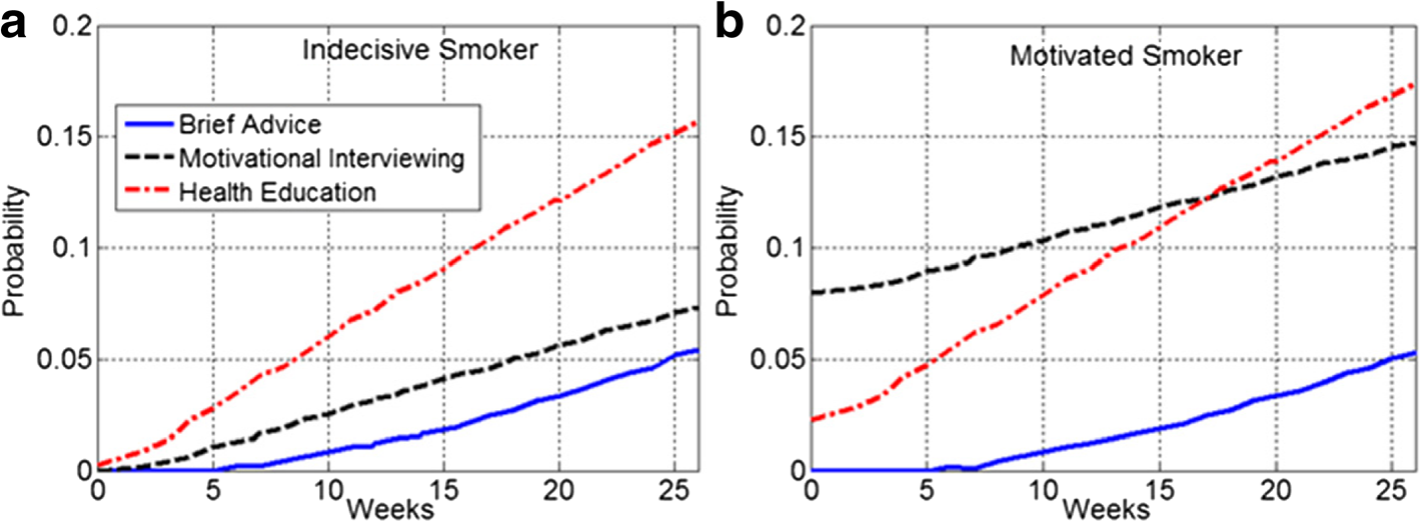
Estimated transition probabilities into the final stage (Former Smoker). **a** Indecisive smokers in HE group had a higher probability to quit. **b** The motivated smokers in the MI group had an overall higher transition probability until week 17

Fig. 4 represents the estimated transition probabilities into the final stage (Former Smoker). As shown in panel (a) the indecisive smokers in HE group had a much higher probability to quit. Panel (b) shows that the motivated smokers in the MI group had a higher transition probability. However, in the HE group, the motivated smokers had slightly higher probabilities to quit from the 17th week onward. The transition probabilities from unmotivated smoker to former smoker were negligible and not reported here.

### Projected performance of the interventions

**Fig. 5:**
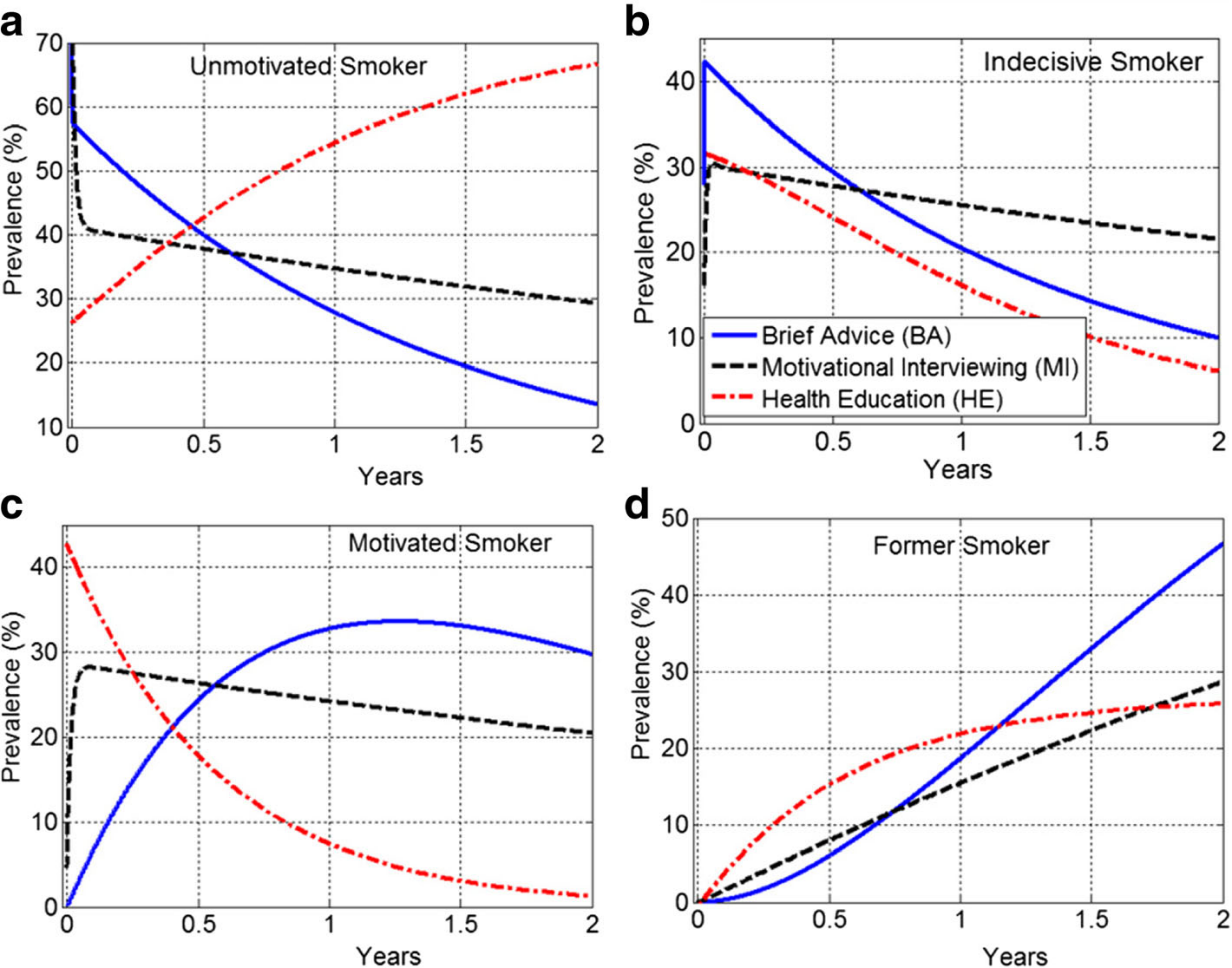
Prevalence of smoker populations projected over 2 years. **a** The prevalence of unmotivated HE participants kept growing and exceeded 60%. **b** There was a sharp decline in the prevalence of indecisive smokers in all three groups. **c** The motivated smokers in HE group reached below 5%. **d** The prevalence of BA former smokers exceeded 45%

The ODE model (4) was employed to predict the temporal changes in the prevalence for 2 years. As shown in panel (b) of Fig. 5, in all three groups of BA, MI and HE the prevalence of indecisive smokers drastically dropped after an initial increase. Furthermore, about 21% of MI participants remained indecisive, whereas BA and HE participants were between 5 to 10% indecisive smokers. Panel (c) shows that more than 20% of MI and BA participants remained motivated. However, the motivated smokers in HE group reached below 5%. Panel (a) shows that more than 60% of HE participants became unmotivated.

Moreover, in contrast to BA and MI groups, the prevalence of unmotivated smokers continuously increased in the HE group. Panel (d) of Fig. 5 indicates that the growth in the population of HE former smokers slowed down dramatically and the prevalence fell below 27%. Also, the prevalence of BA former smokers exceeded 45%. Table 3 provides a summary of the average prevalence in each stage projected over 2 years. The average values were calculated using formula (7). The average prevalence of former smokers was higher in BA group. The HE group had the highest average prevalence of unmotivated smokers. The average prevalence of indecisive and motivated smokers was in the range of 22–27% in all three groups.

**Table 3:** Summary of the average prevalence (%) projected over 2 years. The highest and lowest average prevalence of unmotivated and former smokers belongs to HE

	unmotivated	indecisive	motivated	former smoker
BA	30.5241	22.4779	27.0780	19.9200
MI	37.4583	25.6857	23.5052	13.3508
HE	39.8997	24.0551	25.9601	10.0851

Notes: MI, HE and BA are the abbreviations for Motivational Interviewing, Health Education and Brief Advice, respectively

Table 4 represents the estimated transition rates *d*_ij_ from stage *i* to stage *j* associated with the ODE model (4). For the HE group, the movements were either from motivated to former smoker or motivated to indecisive and thereafter to unmotivated smoker (all other rates of movement are negligible). This may suggest the polarizing effect of HE, where the participants either quit smoking or became highly unmotivated. For the BA group, there was a huge exchange between stages 2 and 1 (indecisive and unmotivated smokers). The rates of movement from these two stages to the last stage were very small. Instead, from these two stages the individuals became motivated and then former smokers. The high frequent exchange between indecisive and unmotivated smokers may suggest that BA was less effectively motivating participants to quit. For the MI group, the movements between the first three stages was fully distributed. Also, the movement from motivated to former smoker was much higher than those from the indecisive and unmotivated smoker groups. This may suggest that MI participants who become motivated to quit were more successful in actually quitting.

**Table 4:** The estimated transition rates *d*_ij_ from stage *i* to stage *j of the ODE model*. The stages unmotivated, indecisive, motivated and former smokers are represented by 1–4, respectively

	unmotivated (1) ↔indecisive (2)			motivated (3)↔ indecisive (2)		
	*d*_12_	*d*_21_	difference	*d*_32_	*d*_23_	difference
BA	3657	2695	962	0.9938	0.0003	0.9935
MI	55.752	37.555	18.1970	13.532	19.135	−5.6030
HE	1.1698	0	1.1698	0	0.6286	−0.6286
	motivated (3) ↔unmotivated (1)			transition rate to former (4)		
	*d*_31_	*d*_13_	difference	*d*_14_	*d*_24_	*d*_34_
BA	0.0003	0.5216	−0.5213	0.00004	0.000006	0.8581
MI	25.941	21.725	4.2160	0.0329	0.0722	0.4692
HE	0	0	0	0	0	1.1147

Notes: MI, HE and BA are the abbreviations for Motivational Interviewing, Health Education and Brief Advice, respectively. The sum of the squared errors related to BA, HE and MI were 3.2381 × 10^−5^, 31.3602 and 2.4501 × 10^−5^ respectively. The precision of the parameter estimate is up to 8 decimals

### Asymptotic analysis of the ODE model

Using the parameter values indicated in Table 4, the asymptotic behavior of the ODE model (4) was investigated. For MI and BA group the trivial equilibrium (*U*^∗^, *I*^∗^, *M*^∗^, *F*^∗^) = (0, 0, 0, 100) is the only equilibrium and it is stable (see part B of the supplementary document and the eigenvalues in Table 5). The average prevalence of former smokers in the MI group is higher than that of BA group. However, in the MI group, it takes almost 4 times longer to converge to the trivial equilibrium (see formula (7) and the last two columns of Table 5). For the HE group, the nontrivial equilibrium (*U*^∗^, *I*^∗^, *M*^∗^, *F*^∗^) = (73.31, 0, 0, 26.68) is stable and the trivial equilibrium is unstable. This suggests that about one out of four participants would become former smokers and the other three would become fully unmotivated. Specifically, less than 27% of the HE participants would become former smokers, which would take about 8.5 years.

**Table 5:** Asymptotic analysis of the ODE solutions. The last column indicates the time needed until all participants become former smokers. This will never happen for HE participants, where less than 27% of them can ever become former smokers

	Eigenvalues (*λ*_1_, *λ*_2_, *λ*_3_)	Equilibrium (*U*^∗^, *I*^∗^, *M*^∗^, *F*^∗^)	Average Prevalence (*U*(*t*), *I*(*t*), *M*(*t*), *F*(*t*))	Time (years)
BA	(−0.9, − 6353.3, − 0.7)	(0, 0, 0, 100)	(6.734, 4.96, 9.798, 78.508)	11.878
MI	(−63.68, −0.17, −110.36)	(0, 0, 0, 100)	(6.293, 4.526, 4.246, 84.935)	40.664
HE	(−1.743, −1.17, 0)	(73.31, 0, 0, 26.68)	(66.762, 5.252, 3.608, 24.378)	Infinity

Notes: MI, HE and BA are the abbreviations for Motivational Interviewing, Health Education and Brief Advice, respectively. For HE group 8.445 years is needed to reach that equilibrium. The notations U^∗^, I^∗^, M^∗^and F^∗^ represent the prevalence of indecisive, motivated, unmotivated and former smokers at the equilibrium, respectively

## Discussion

Despite smoking rates declining from 41.9 to 19.3% in the past 50 years, tobacco use remains the leading cause of preventable morbidity and mortality in the United States [28]. A concerning finding is that the rate of smoking decrease has dramatically slowed in the most recent decade [29]. While great strides have been made in developing effective pharmacological and behavioral interventions to help smokers who are motivated to try to quit, the vast majority of current smokers (approximately 80%) are not willing or ready to quit [13, 30]. Thus, there is a great need for clinical interventions that enhance smokers’ motivation to try and quit. An important outcome of the present study is that, at the same time that several HE participants successfully quit smoking (see panel (d) of Figs. 2 and 5), many others lost their motivation to quit or stopped their quit attempts (see panels (a) and (c) of Figs. 2 and 5). 

One key to understanding a broader range of intervention effects is the method used to analyze the outcomes. Whereas in RCT’s it has been traditional to compare mean effects between treatment and control to establish treatment effects, these kinds of analyses can mask intervention effects on subsets of participants. To address this issue, secondary analyses often examine the effects of interactions between participant baseline characteristics and treatment. But these analyses fail to consider the full range of possible motivational outcomes for *all* of the participants. Additionally, smoking cessation is gradually being documented as a dynamic practice where people abandon, relapse, and quit again, often with recurrent cycles over years [7].

The multi-state modeling [11, 12] is a convenient way to describe a continuous random time process. The MSM is a freely available package, which permits a longitudinal data to be fitted with a general MC model. The MSM package for R allows the simulation of disease progression and reveals the effects of different interventions on treatment results.

A potential drawback of the MC model used in this study is that “former smoker” is considered as an absorbing state. However, two cases in this study had a relapse in MI and HE groups, which indicates that the “absorbing” assumption may not be true especially in a larger population. Nevertheless, the relapse in MI and HE groups were extremely low (2 out of 177 transitions in MI group and 2 out of 184 transitions in HE group; see Addditional file 1 Table A1 ). Therefore, the impact of relapse is more likely to be negligible in the present study. More complicated MC models can be used to study the effects of relapse (see for example [10]).

In the present study data of 15.6, 14.71 and 10.8% of participants in BA, MI and HE groups respectively were excluded due to missing values. We noted that almost all of these participants were present only at the beginning of the study and there was no follow up data values. Further analysis of the data with missing values did not indicate any clear pattern and therefore excluded from the study. There are several imputation-based strategies for clinical trial data with missing values [10, 31–33]. However, the results of models fitted to data with missing values should always be interpreted with extra caution.

As a two-step MC-ODE modeling process proposed in this study, it was demonstrated that the prevalence of HE motivated smokers was drastically decreased and simultaneously there was an incline in the prevalence of unmotivated smokers (see Fig. 2). Although HE had a greater efficacy compared to MI and BA (i.e., the highest smoking cessation rate), the results revealed the shortcomings of HE intervention with respect to the effectiveness (see Fig. 3). Using an ODE modeling approach, the results of the MC analysis were projected into 2 years. It was found that the impacts of HE on the 7-day point-prevalence smoking abstinence substantially diminished over time. The 2-year ODE model projections (see Fig. 4) show that the prevalence of motivated smokers in the HE group may fall below 5%. The prevalence of HE former smokers can reach an equilibrium of 26.7%, where the prevalence of both BA and MI former smokers may exceed this equilibrium.

## Conclusion

In conclusion, the methodology proposed in this paper is a two-step process, which benefits from the robustness of both probabilistic and deterministic modeling via MC and ODE models, respectively. The successive MC-ODE modeling can be used in a variety of case studies, where the related data has very few observations over time. Here, the method was employed to determine and compare the relative effectiveness of smoking cession interventions, yielding predictive insights regarding the temporal characteristics and capabilities of these interventions during the study period and beyond.

## Additional file

Additional file 1: Details of the MC and ODE Model Analysis. (DOCX 30 kb)

## Abbreviations

BA: Brief Advice
HE: Health Education
MC: Markov Chain
MI: Motivational Interviewing
ODE: Ordinary Differential Equations
RCT’s: Randomized controlled trials
