# Supplementary material for: Effectiveness of motivational interviewing, health education and brief advice in a population of smokers who are not ready to quit
Source: BMC Med Res Methodol. 2018 Jun 13;18:52. doi: 10.1186/s12874-018-0511-0 (PMC5998452; doi:10.1186/s12874-018-0511-0)
Supplement: Supplementary file 1 — Details of the MC and ODE Model Analysis. (DOCX 30 kb) [file 12874_2018_511_MOESM1_ESM.docx]

**Additional file**

**Part A: Details of the MC Model Analysis**

For BA, MI and HE, the dataset consisted of 86, 177 and 184 transitions between the four stages, respectively. Table A1 provides a summary of the observed transitions from one stage to another.

| \| **Table S1.** Summary of the observed number of transitions between stages 1-4. \| \| \| \| \| \| \| \| \| \| --- \| --- \| --- \| --- \| --- \| --- \| --- \| --- \| --- \| \|  \| \| \| \| **To** \| \| \| \| \| \| **From** \| 1 \| \| \| \| 2 \| 3 \| 4 \| 99 \| \|  \| \| **Brief Advice (BA)** \| \| \| \| \| \| \| \| 1 \| 12 \| \| \| \| 6 \| 33 \| 0 \| 0 \| \| 2 \| 16 \| \| \| \| 6 \| 5 \| 1 \| 0 \| \| 3 \| 0 \| \| \| \| 5 \| 0 \| 2 \| 0 \| \| 4 \| 0 \| \| \| \| 0 \| 0 \| 0 \| 0 \| \|  \| \| \| **Motivational Interviewing (MI)** \| \| \| \| \| \| \| 1 \| 27 \| \| \| \| 16 \| 59 \| 3 \| 0 \| \| 2 \| 16 \| \| \| \| 14 \| 6 \| 1 \| 0 \| \| 3 \| 7 \| \| \| \| 18 \| 3 \| 3 \| 0 \| \| 4 \| 0 \| \| \| \| 0 \| 0 \| 2 \| 2 \| \|  \| \| \| **Health Education (HE)** \| \| \| \| \| \| \| 1 \| 17 \| \| \| \| 30 \| 45 \| 5 \| 0 \| \| 2 \| 23 \| \| \| \| 16 \| 5 \| 2 \| 0 \| \| 3 \| 13 \| \| \| \| 8 \| 3 \| 9 \| 0 \| \| 4 \| 0 \| \| \| \| 0 \| 0 \| 6 \| 2 \| |
| --- | --- | --- | --- | --- | --- | --- | --- | --- | --- | --- | --- | --- | --- | --- | --- | --- | --- | --- | --- | --- | --- | --- | --- | --- | --- | --- | --- | --- | --- | --- | --- | --- | --- | --- | --- | --- | --- | --- | --- | --- | --- | --- | --- | --- | --- | --- | --- | --- | --- | --- | --- | --- | --- | --- | --- | --- | --- | --- | --- | --- | --- | --- | --- | --- | --- | --- | --- | --- | --- | --- | --- | --- | --- | --- | --- | --- | --- | --- | --- | --- | --- | --- | --- | --- | --- | --- | --- | --- | --- | --- | --- | --- | --- | --- | --- | --- | --- | --- | --- | --- | --- | --- | --- | --- | --- | --- | --- | --- | --- | --- | --- | --- | --- | --- | --- | --- | --- | --- | --- | --- | --- | --- | --- | --- | --- | --- | --- | --- | --- | --- | --- | --- | --- | --- | --- | --- | --- | --- | --- | --- | --- | --- | --- | --- | --- | --- | --- | --- | --- | --- | --- | --- | --- | --- | --- | --- | --- | --- | --- | --- | --- | --- |

Notes: Stages 1-4 represent unmotivated, indecisive, motived, and former smokers, respectively. Stage 99 represents the censored cases (i.e., these cases were excluded from the MC modeling)

| **Table S2.** Estimated transition intensity *entry of matrix* $Q$ with 95% confidence intervals. | | | |
| --- | --- | --- | --- |
|  | **Health Education** | **Motivational Interviewing** | **Brief Advice** |
|  | -0.0158 (-0.0211, -0.011) | -0.1188 (-1.527, -0.0092) | -0.0096 (-0.0159, -0.0058) |
|  | 0 (0, 0 ) | 0 (0, 0 ) | 0 (0, 0 ) |
|  | -0.0323 (-0.0646, -0.016) | -0.5315 (-58.15, -0.0049) | -0.0042 (-0.013, -0.0013) |
|  | 0.0042 (0.0003, 0.055) | 0.0001 (0, 3.14) | 0.0042 (0.0013, 0.013) |
|  | 0.0158 (0.0117, 0.021) | 0.0875 (0.0739, 0.1036) | 0.0018 (0.0004, 0.0075) |
|  | -0.0158 (-0.0211, -0.011) | -0.1188 (-1.527, -0.0092) | -0.0096 (-0.0159, -0.0058) |
|  | 0 (0, 12.902) | 0.0312 (0, 404.5) | 0.0078 (0.0041, 0.0147) |
|  | 0 (0, 423.2) | 0.0001 (0, 1.186) | 0 (0, 128.55) |
|  | 0.0189 (0.0073, 0.049) | 0.4008 (0.0009, 176.775) | 0.006 (0.0027, 0.0135) |
|  | 0.0053 (0.0025, 0.0112) | 0.1597 (0.0136, 1.8818) | 0.0059 (0.0023, 0.0151) |
|  | -0.026 (-0.0613, -0.0109) | -0.563 (-47.325, -0.0067) | -0.0119 (-0.022, -0.0064) |
|  | 0.0017 (0, 1.1125) | 0.0025 (0.0006, 0.0098) | 0 (0, 130.625) |

*Notes: Stages 1-4 are unmotivated, indecisive, motivated, and former smokers. The entry of matrix* $Q$ *is the intensity of moving from stage i to stage j.*

**Part B: Details of the ODE Model Analysis**

The ODE model (4) can be rewritten in matrix form

$X^{'}\left( t \right)=AX\left( t \right),$ (B1)

where

$A=\left[ \begin{matrix} -{(d}_{12}+d_{13}+d_{14}) & d_{21} & d_{31} \\ d_{12} & -{(d}_{21}+d_{23}+d_{24}) & d_{32} \\ d_{13} & d_{23} & -(d_{31}+d_{32}+d_{34}) \end{matrix} \right],$ (B2)

and

$X\left( t \right)=\left[ \begin{matrix} U\left( t \right) \\ I\left( t \right) \\ M\left( t \right) \end{matrix} \right]$ (B3)

Using the fundamental theorem for linear systems [29] the solution of the initial value problem (IVP)

$\left\{ \begin{matrix} X^{'}\left( t \right)=AX(t) \\ X\left( 0 \right)=X_{0} \end{matrix} \right.,$ (B4)

is given by

$X\left( t \right)=\exp\left( At \right)X_{0},$ (B5)

When matrix A is diagonalizable the solution of IVP is given by

$X\left( t \right)=P exp\left( \Lambda t \right) P^{-1}X_{0}$, $,$ (B6)

where $\Lambda$ is a diagonal matrix, whose element are the eigenvalues of A and P is an invertible matrix whose columns are the eigenvectors of A. See Table 4 for the specific eigenvalues related to each intervention. For MI and BA the trivial solution of $X^{'}\left( t \right)=AX\left( t \right)$ is stable and therefore $X\left( t \right)\to0 as t\to\infty.$ For HE, the trivial equilibrium is unstable but the determinant of A is zero and there are infinitely many equilibria, where the solutions converge to one of them (see Table 4). The rate of convergence is governed by the spectral radius of A, denoted by $\rho\left( A \right)$. As indicated in the last column of Table 5, the convergence to the trivial equilibrium is the fastest in the BA group.
